# Trapping a salt-dependent unfolding intermediate of the marginally stable protein Yfh1

**DOI:** 10.3389/fmolb.2014.00013

**Published:** 2014-09-30

**Authors:** Bartolomé Vilanova, Domenico Sanfelice, Gabriel Martorell, Piero A. Temussi, Annalisa Pastore

**Affiliations:** ^1^Departament de Química, Universitat de les Illes BalearsPalma de Mallorca, Spain; ^2^Molecular Structure Division, National Institute for Medical ResearchLondon, UK; ^3^Serveis Científico-Tècnics, Universitat de les Illes BalearsPalma de Mallorca, Spain; ^4^Department of Chemistry, Università di Napoli Federico IINapoli, Italy

**Keywords:** cold denaturation, frataxin, metallo proteins, NMR, thermodynamic stability

## Abstract

Yfh1, the yeast ortholog of frataxin, is a protein of limited thermodynamic stability which undergoes cold denaturation at temperatures above the water freezing point. We have previously demonstrated that its stability is strongly dependent on ionic strength and that monovalent or divalent cations are able to considerably stabilize the fold. Here, we present a study of the folded state and of the structural determinants that lead to the strong salt dependence. We demonstrate by nuclear magnetic resonance that, at room temperature, Yfh1 exists as an equilibrium mixture of a folded species and a folding intermediate in slow exchange equilibrium. The equilibrium completely shifts in favor of the folded species by the addition of even small concentrations of salt. We demonstrate that Yfh1 is destabilized by a localized energetic frustration arising from an “electrostatic hinge” made of negatively charged residues mapped in the β-sheet. Salt interactions at this site have a “frustration-relieving” effect. We discuss the consequences of our findings for the function of Yfh1 and for our understanding of protein folding stability.

## Introduction

The yeast protein Yfh1 is a member of the highly conserved frataxin family that is present in most organisms from bacteria to primates (Pandolfo and Pastore, 2009). Yfh1 is a particularly fascinating protein amongst the frataxin orthologs. As all frataxins, it consists of a globular domain with an αβ fold that is conserved in all species. However, despite the high degree of conservation, Yfh1 is thermodynamically highly unstable at variance with other prokaryotic and eukaryotic members of the family, having the middle of its folding/unfolding transition around 35°C, i.e., close to the optimal growth temperature of yeast (Adinolfi et al., 2004). It also undergoes another transition at low temperature, around 5°C, being one of the very few known examples of a protein that undergoes cold denaturation at temperatures above water freezing point (Pastore et al., 2007). We have previously characterized in detail both the cold and heat unfolded states of Yfh1 and showed that they have similar residual secondary structure elements reminiscent of the architecture of the folded state, albeit with slightly different compactness (Adrover et al., 2012). We identified in the exquisite sensitivity of Yfh1 to even small concentrations of salts, best if divalent or trivalent cations, an important factor to stabilize both unfolded states (Nair et al., 2004; Pastore et al., 2007; Sanfelice et al., 2014).

Thanks to the possibility of detecting both melting points, we reconstructed the whole stability curve that is, the plot of ΔG as a function of temperature. The bell-shaped curve is narrow with a maximal stability in the range 18–23°C (Pastore et al., 2007; Martin et al., 2008; Sanfelice et al., 2014). By simple fitting, the curve allows to extract the thermodynamic parameters of the unfolding transitions and to measure the percentage of folded protein at the maximal stability temperature (Pastore et al., 2007; Martin et al., 2008; Sanfelice et al., 2014). This is only around 60–70% (Pastore et al., 2007) a value which cannot solely be explained by the presence of an N-terminal mitochondrial import signal (He et al., 2004; Karlberg et al., 2006). This observation suggests the possibility that Yfh1 exists in a partially unfolded state also at room temperature.

To explore this possibility, we focused on studying Yfh1 at room temperature to ultimately understand the structural determinants that make Yfh1 such a low stability protein and the bases of its salt sensitivity. Using NMR, we show here that Yfh1 exists in equilibrium between a folded species and a folding intermediate that has features in common with the cold and heat unfolded states. Even a small increase of the ionic strength is able to shift the equilibrium toward the folded species in agreement with the unusual sensitivity of Yfh1 to salts. The presence of salts strongly affects a negatively charged patch on the β-sheet indicating that this is the main source of the energetic frustration of the Yfh1 fold. Our results strongly suggest Yfh1 to be another representative of metamorphic proteins whose fold depends on ionic strength.

## Materials and methods

### Sample preparation

Recombinant *S. cerevisiae* Yfh1 was produced as previously described in details (Adinolfi et al., 2002; He et al., 2004) In short, the protein was expressed in *Escherichia coli* BL21-(DE3) cells grown at 37°C, induced in 0.5 mM IPTG for 2 h. Cells were harvested by centrifugation and re-suspended in Tris-HCl buffer containing a complete EDTA protease inhibitor cocktail tablet (Roche) and lysed by 5 cycles of sonication. The soluble, overexpressed protein was purified by two ammonium sulfate precipitation steps with a 40% cut to precipitate contaminating proteins and a 65% cut to precipitate Yfh1. After dialysis, the protein was subjected to anion exchange chromatography using a Pharmacia Q-Sepharose column with a gradient to 1 M NaCl, followed by a Pharmacia phenyl-Sepharose column with a decreasing 1 M ammonium sulfate gradient. EDTA and salts were removed by dialysis prior to concentration of the protein. ^15^N-labeled and ^15^N,^13^C double-labeled samples were produced by growing the bacteria in minimal medium using ammonium sulfate and glucose as the sole source of nitrogen and carbon. Final sample was stored in 20 mM HEPES 2 mM DTT pH 7.0.

### NMR spectroscopy

^15^N- and ^15^N,^13^C-labeled Yhf1 samples used for NMR studies (~0.5 mM) were in 20 mM HEPES at pH 7.0 and 2 mM DTT, containing 5% (v/v) D_2_O. NaCl was added prior the experiments from a stock (5 M) to a final concentration of 100 mM. Multidimensional NMR experiments were carried out at 298 K on a Bruker Avance spectrometer operating at 600 and 700 MHz proton frequency and equipped with triple-resonance cryo-probe. The assignment of the backbone resonances of Yfh1 were accomplished by recording and analyzing HNCACB, CBCA(CO)NH, HNCO, and HBHACONH triple resonance datasets (Sattler et al., 1999). Data processing and analysis were performed using the nmrPipe (Delaglio et al., 1995), CARA (Keller, 2004), and NMRviewJ (Johnson and Blevins, 1994) software packages, respectively. Water was suppressed by the Watergate pulse sequence (Piotto et al., 1992). Proton chemical shifts were referenced to the water signal fixed at 4.7 ppm. ^13^C and ^15^N chemical shifts were referenced indirectly using the ^1^H, X frequency ratios of the zero-point (Wishart et al., 1995). Secondary structure propensity (SSP) scores were calculated using the SSP software (Marsh et al., 2006). ψ and φ angles (Figure S1) were evaluated using Talos+ (Shen et al., 2009). The assigned chemical shifts of Yhf1 at 298 K are deposited in BioMagResBank (http://www.bmrb.wisc.edu) under accession number 19991.

### Rosetta modeling

Structures of Yfh1 were calculated using chemical shift-based Rosetta (Rosetta 3.4) using a standard protocol (Ramelot et al., 2009) and incorporating backbone N, H^N^, C, C^α^, H^α^, and C^β^ chemical shifts. After initial testing, the first 10 residues (from M51 to V61) of the intrinsically unfolded N-terminus were excluded from the calculation. The calculations generated 10000 all-atom models, and the 1000 low-energy models were extracted and further rescored against chemical shifts. The final 10 lowest energy structures were chosen as representative of the calculation. Pdb 2ga5 and 2fql were excluded from the fragment search in order to have an unbiased calculation.

## Results

### At room temperature the folded state is in equilibrium with a folding intermediate

**Figure 1:**
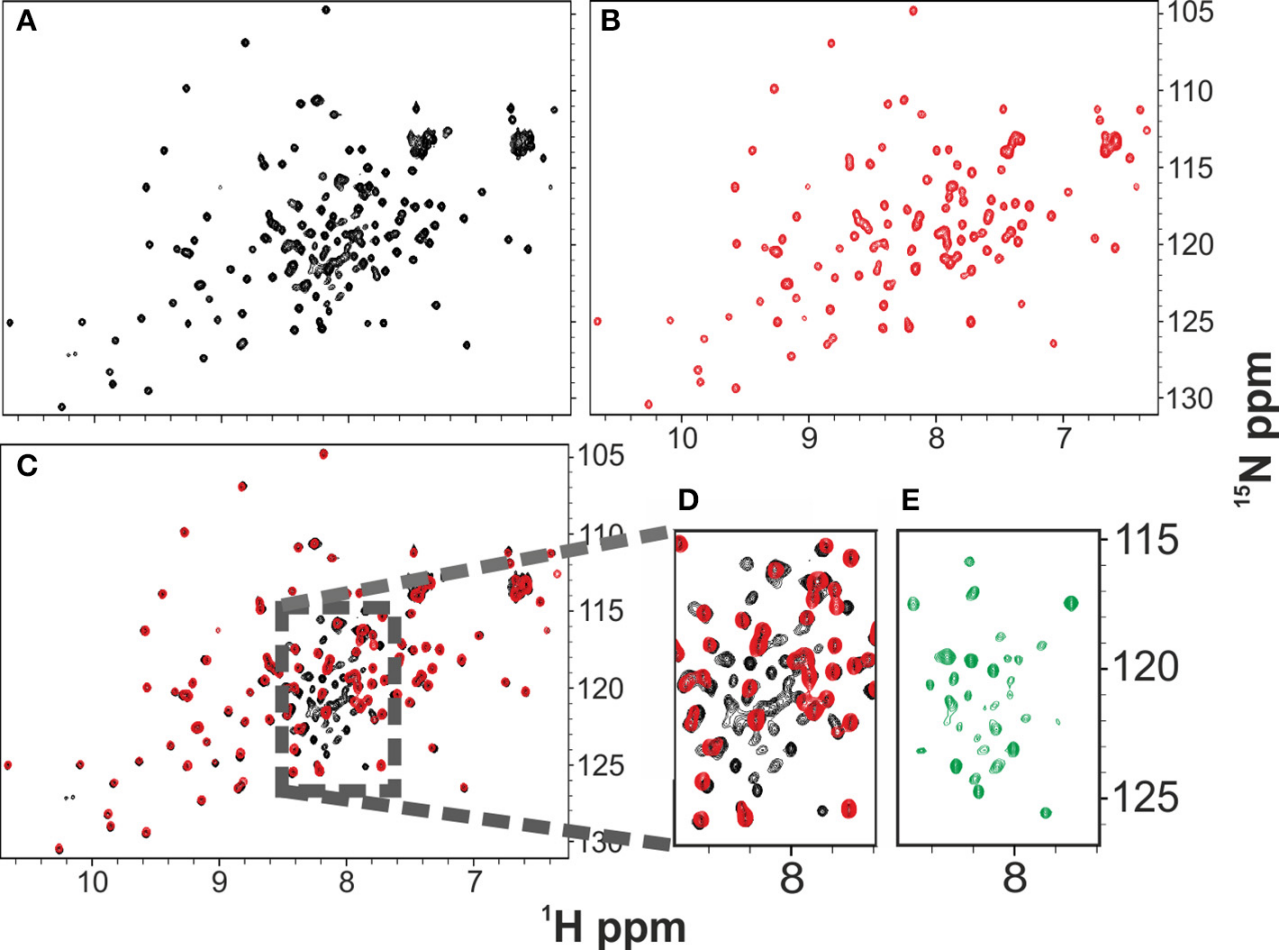
**Comparison of the ^1^H ^15^N HSQC NMR spectra of Yfh1 in the presence (red) and in the absence (black) of salt**. NMR experiments were acquired at 25°C. **(A)** HSQC spectrum of freshly produced Yfh1 in 20 mM HEPES buffer, pH 7.0. **(B)** HSQC spectrum of Yfh1 in 20 mM HEPES buffer with the addition of 100 mM NaCl, pH 7.0. **(C)** Overlay of spectra **(A,B)** showing that many peaks of the central region (typical for unfolded protein peaks) disappear after salt addition. **(D)** Zoom of the central region between 8.5 and 7.5 ppm. **(E)** Zoom of the central region between 8.5 and 7.5 ppm after subtraction of the red peaks: the remaining peaks coincide with those previously assigned to the unfolded species (Adrover et al., 2010, 2012).

The HSQC NMR spectrum of freshly produced and desalted Yfh1 at 23°C is overall well dispersed and typical of a well folded protein (Figure 1A, black). This is the temperature of maximum stability (*Ts*) at these solution conditions, as extracted from its full stability curve (Sanfelice et al., 2014). However, closer inspection reveals the presence of more resonances than those expected for the sequence. In addition to the main peaks, several less intense resonances are observed, which are in the region of the spectrum expected for resonances of residues in a random coil conformation. The presence of these peaks was observed in independently produced batches of protein, suggesting that they account either for reproducible contaminants or for minor populations of the same protein.

### Salt stabilizes the folded state of Yfh1

To test the hypothesis that the additional resonances could come from an unfolded or partially unfolded intermediate of Yfh1 in co-presence with the folded one, we added salt since we had earlier realized that salts have an appreciable effect on the protein stability (Adinolfi et al., 2004). A recent quantitative study on the effect of several salts revealed that divalent cations bind specifically at micromolar concentrations, greatly stabilizing the folded species, but even buffers containing monovalent cations induce sizeable stabilization at a relatively modest ionic strength (Sanfelice et al., 2014). By addition of 100 mM NaCl, we observed complete and quantitative disappearance of the additional resonances and a minor shift of some resonances according to the change of experimental conditions (Figures 1B–E). The effect is fully reversible: desalting leads to reappearance of the extra peaks (Figure 1C).

**Figure 2:**
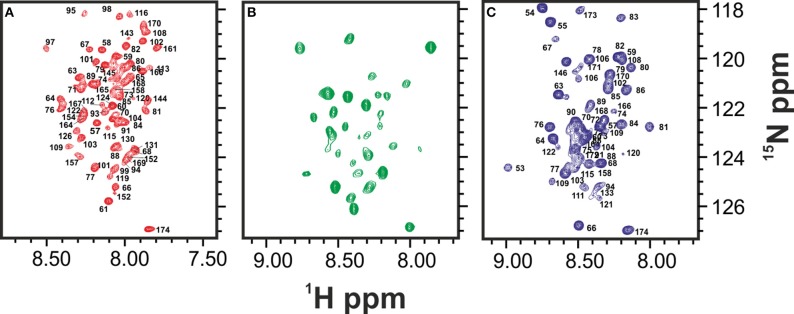
**Comparison of the HSQCs of the unfolded species of Yfh1 at high (red), low (blue), and room temperature (green). (A)** HSQC spectrum of Yfh1 in 20 mM HEPES buffer, pH 7.0 at 45°C (Adrover et al., 2012). **(B)** HSQC spectrum of Yfh1 in 20 mM HEPES, pH 7.0 at 25°C. **(C)** HSQC spectrum of Yfh1 in 20 mM HEPES buffer, pH 7.0 at 0°C (Adrover et al., 2010).

These results strongly suggest that the folded and the intermediate forms are in mutual exchange so that the latter species disappears when the protein is stabilized. Comparison among the spectra at 0, 23, and 45°C suggests an overall similarity of the chemical shifts. There is not, of course, a precise coincidence, partly because of temperature dependence of all resonances (Figure 2) (Adrover et al., 2010). The number of resonances observed at 25°C for the intermediate is overall a relatively small number (ca. 30 resonances) suggesting a partial unfolding only in this region.

### Spectral assignment provides important information on the folded state

**Figure 3:**
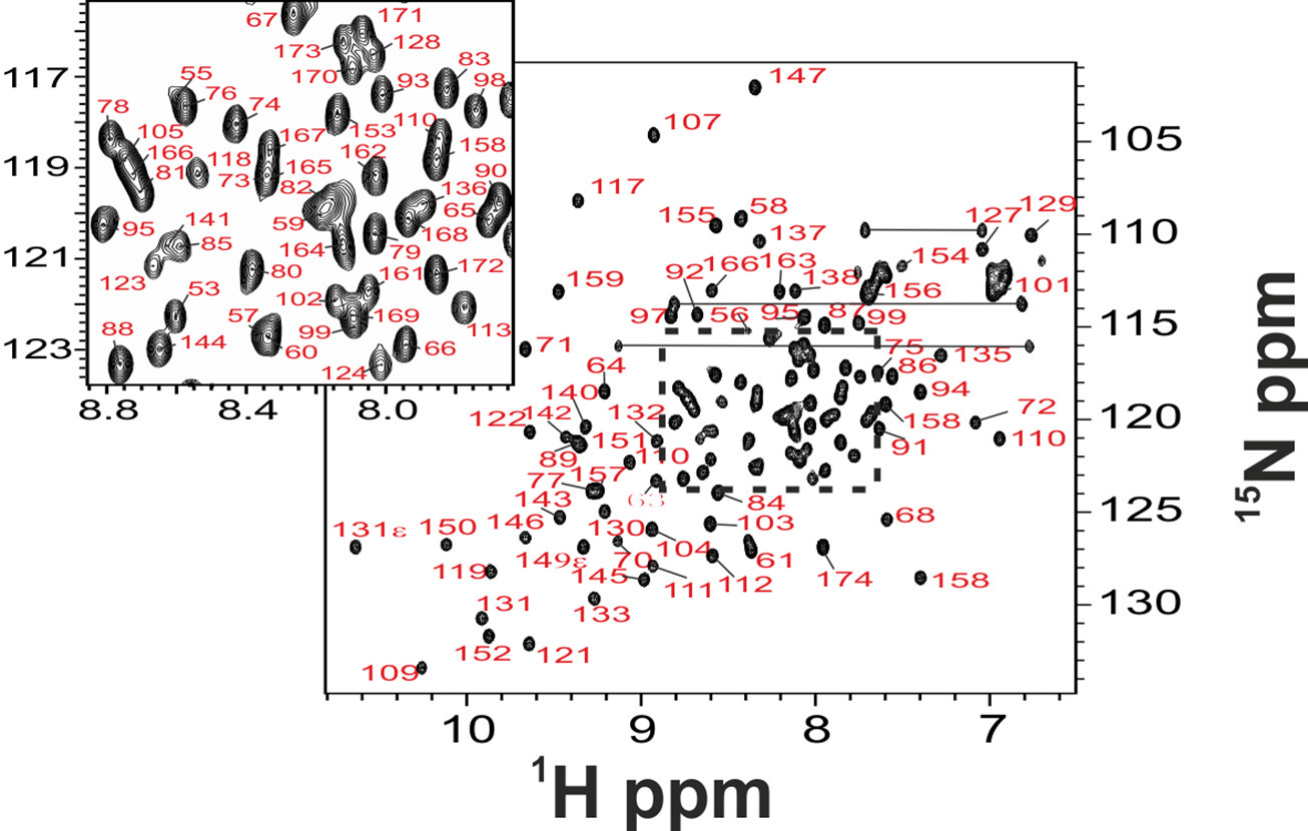
**Full backbone NMR assignment of folded Yfh1. NMR experiments on Yfh1, in 20 mM HEPES buffer with the addition of 100 mM NaCl, pH 7.0, were acquired at 25°C**. The inset shows assignment details in the region where there is maximum overlap of peaks typical of the unfolded species.

To identify the residues in exchange, we tried to assign the resonances of the minor species. For consistency with previous NMR studies (Cook et al., 2006) the assignment was performed at 25°C, a condition only marginally different from *T_S_*. However, we realized immediately that the available assignment of the spectrum of folded Yfh1 (BMRB accession code: 6356) may be partially incorrect as it traces resonances both from the folded species and the folding intermediate. Exploiting recent findings on the influence of salts on the stability of Yfh1 (Sanfelice et al., 2014), we assigned the spectrum anew both at nominal “zero ionic strength” and at moderate ionic strength (100 mM NaCl) (Figure 3).

**Figure 4:**
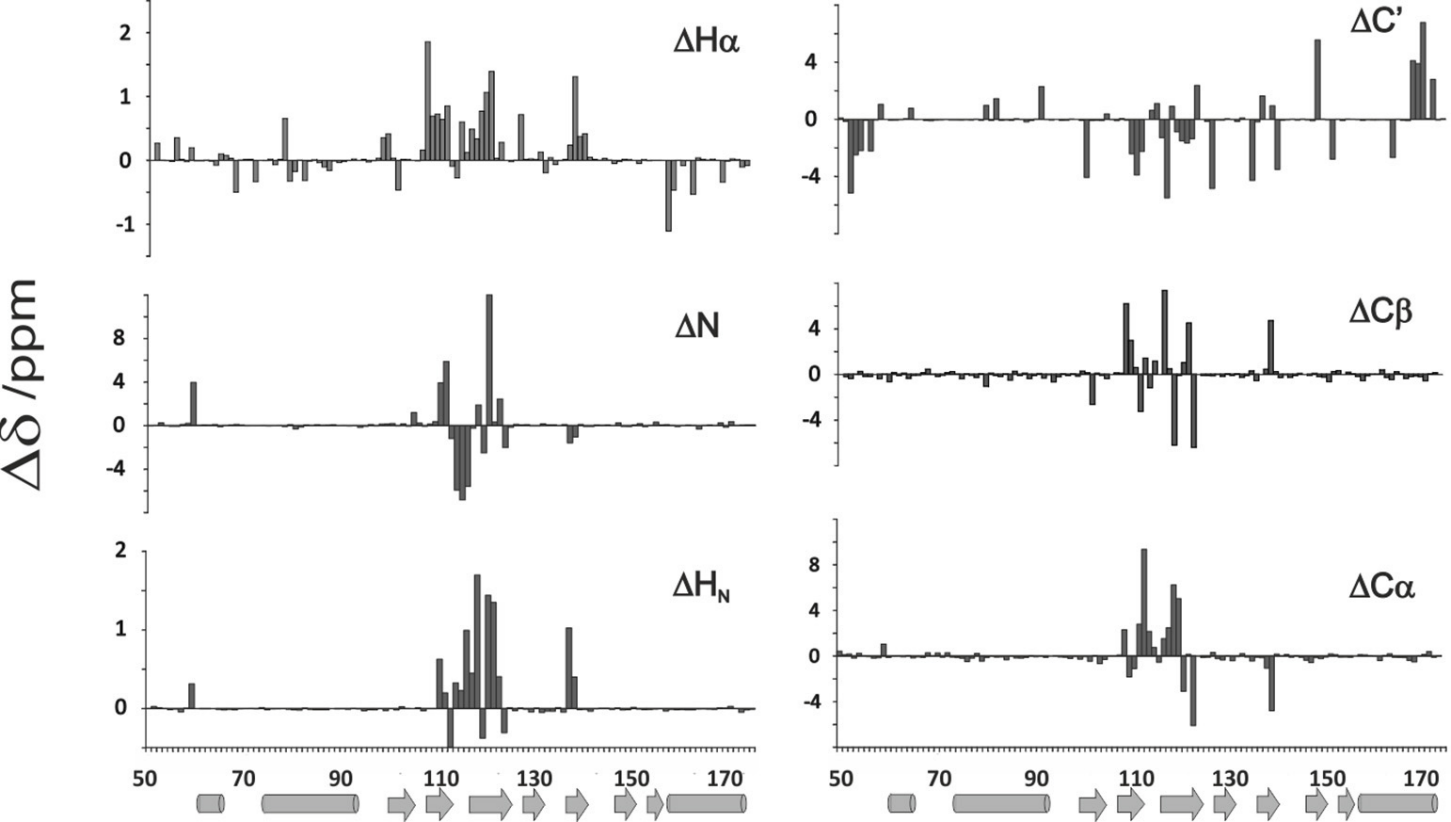
**Comparison of newly assigned chemical shifts with those previously assigned to yeast frataxin (He et al., 2004; Cook et al., 2006) (BMRB accession code: 6356)**. The bar graphs report differences between the two assignments. Salts have a strong influence on the residues going from 113 to 125 of the matured Yfh1. Percentage of residues presenting a δ /ppm absolute variation larger than 0.2 (Δδ ppm) are 13.0% HN, 12.2%N, 26.8% Hα, 16.3% Cα, 17.1% Cβ, and 28.5% C'.

The new assignment differs from the old one by 12% for N, up to 31% for Hα (see also Figure 4) and is virtually complete having assigned also the aromatic side chains (but for H106) which were missing in the previous assignment. Interestingly, the major differences between the two assignments are observed in the middle of the sequence, between residues 113 and 125 which correspond to a β-sheet region (Figure 4). These residues were correctly traced in the sequence but incorrectly assigned because they come from the same residues but in the unstructured form.

### Structure determination of Yfh1 in solution

**Figure 5:**
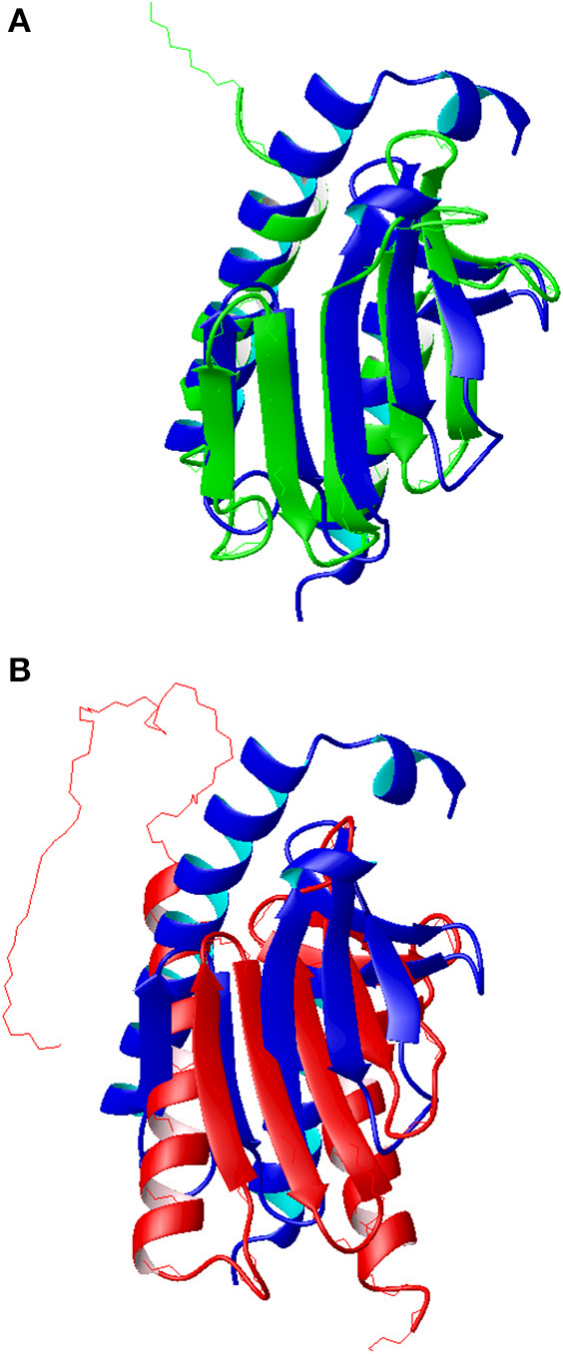
**Comparison of the molecular models of Yfh1. (A)** Superposition of the Rosetta model (blue ribbon) with the x-ray structure of a mutant Yfh1 (green ribbon, pdb id 2fql). **(B)** Superposition of the Rosetta model (blue ribbon) with the previous NMR structure (red ribbon, pdb id 2ga5). Molecular models, represented as ribbons, were built with MOLMOL (Koradi et al., 1996).

As a validation of the newly determined assignment, we used the chemical shifts to model the structure of Yfh1 using the CS-Rosetta software (Qian et al., 2007; Shen et al., 2008; Ramelot et al., 2009). This method is based on an empirically optimized procedure that selects protein fragments from the Protein Data Bank, in conjunction with the standard ROSETTA Monte Carlo assembly. It allows the structural use of chemical shifts even in the absence of NMR restraints, resulting in significant improvements in structure quality and root mean square deviations (r.m.s.d.) with respect to the crystal structures. When applied to the new assignment of Yfh1 the method converged effortlessly into a structure that shows all the features of the frataxin fold having two helices packing against a five strands β-sheet (Figure 5). Convergence improves by cutting off the first 10 residues which seems to be unfolded. Interestingly, the Rosetta software did not find convergence when using the previous assignment (Cook et al., 2006).

The r.m.s.d. with the crystallographic structure (pdb id 2fql) is 2.05 Å (Figure 5A), whereas the corresponding r.m.s.d. with the previous NMR structure (pdb id 2ga5) is 3.05 Å (Figure 5B). The major differences between the published structures and the Rosetta model, besides the different orientation of secondary structure elements in the previous NMR structure, are located at the N terminal. The NMR structure and the Rosetta model have same length of helix 1 while the crystal structure is two turns shorter, partly because it refers to a Y73A mutant designed to render the N-terminal more flexible. The solution structure has also a 16aa long unstructured N terminal that is anchored to helix one; in the other two structures this region, although very flexible, presents a small helix.

### Characterization of the folding intermediate

**Figure 6:**
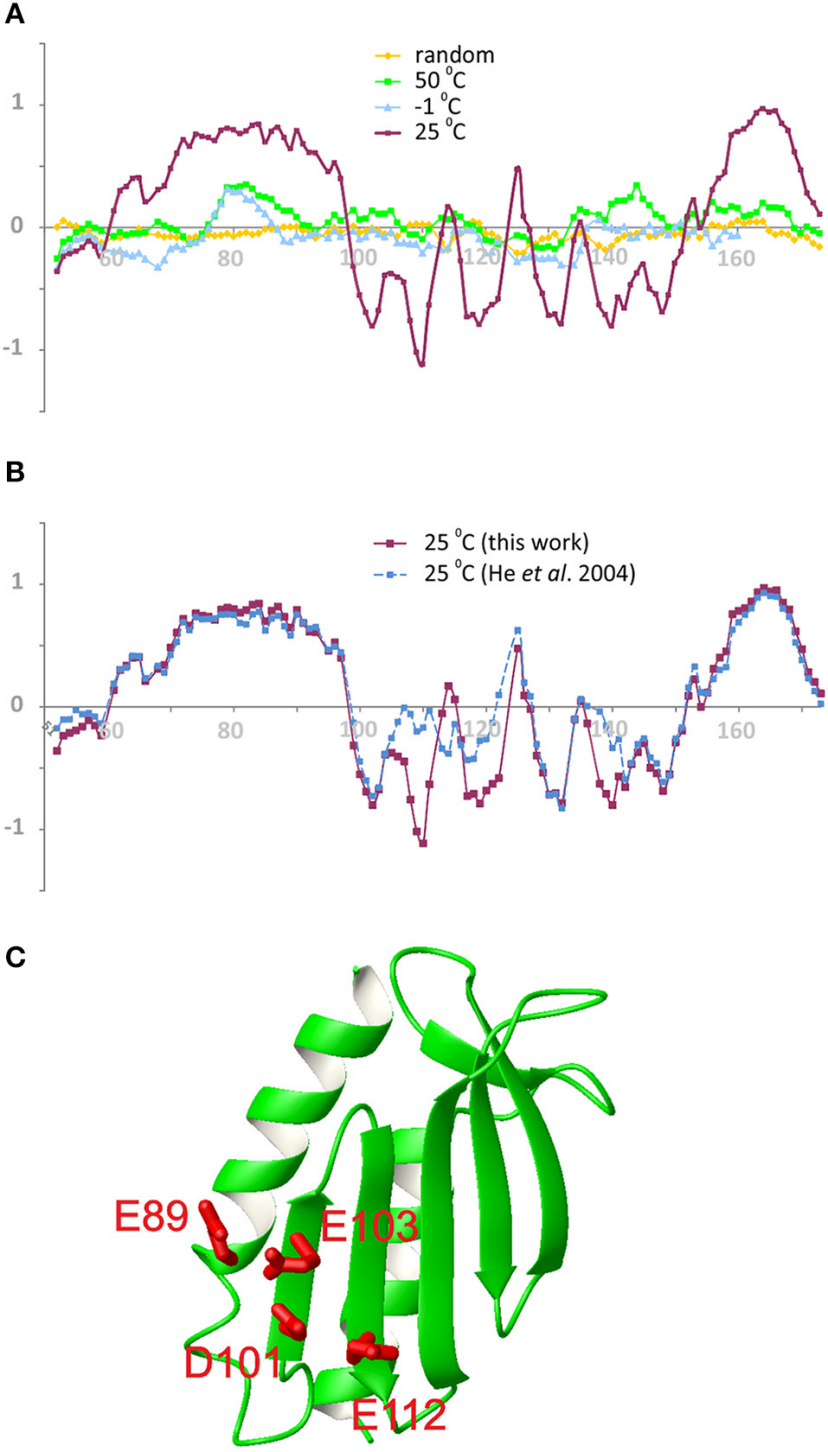
**SSP values along the sequence of Yfh1. (A)** Comparison of compounded chemical shifts of the folded species (magenta) with those of typical random coil (yellow) and with those of the unfolded species at −1°C (cyan) and 50°C (green). **(B)** The new assignment (magenta) compared with the existing assignment (blue). Negative values in the critical region between 107 and 121 indicate that the assignment in the presence of salt is compatible with the presence of β-strands in the central region of Yfh1, while previous assignment is not. **(C)** View of the molecular model of Yfh1 including of the main residues defining the electrostatic hinge (E89, D101, E103, E112). Residue numbers are those of pdb id:2FQL. The molecular model was generated by MOLMOL (Koradi et al., 1996).

To characterize further the unfolded species and understand its degree of unfolding, we used the SSP indices, which combine different chemical shifts into a single secondary structure propensity score (Marsh et al., 2006) (Figure 6A). The positive and negative values ought to correlate with the helical and β-sheet elements respectively, whereas values around zero indicate a random coil conformation. The values of the folded species correlate well with what observed in the frataxin family. The chemical shifts of the folding intermediate have, instead, a clear departure from the secondary structure observed for the folded species between 107 and 121 (Figure 6B). The values are closer to the random coil values expected for this sequence. Interestingly, the SSP profile in this region is more similar to that observed in both the high and low temperature unfolded states (Adrover et al., 2010, 2012).

**Figure 7:**
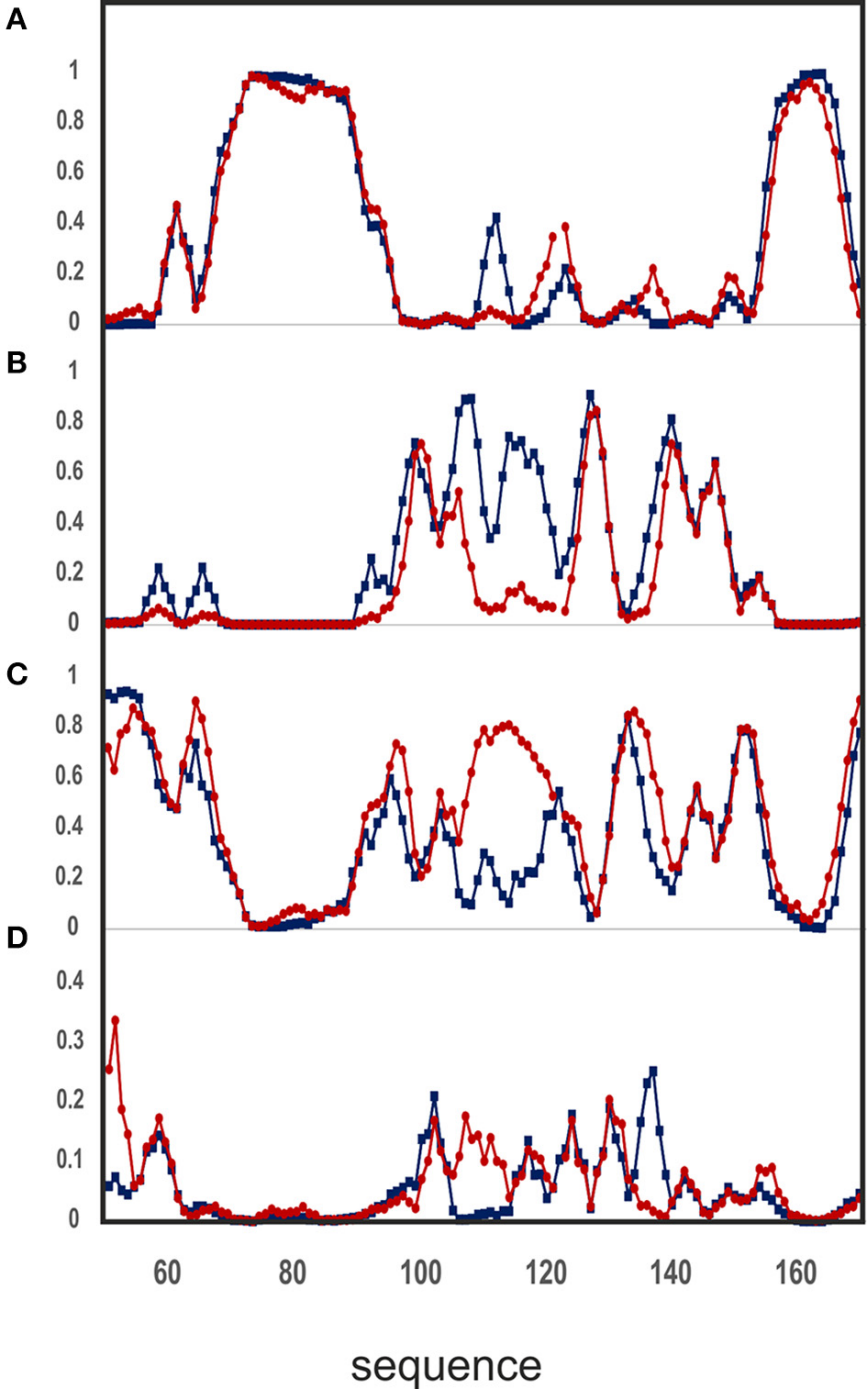
**Analysis of chemical shifts according to the δ2D software, version 1.2**. Comparison of the NMR assignment of the backbone atoms for Yfh1 from this work (blue) and that previously reported (BMRB accession code: 6356/red) against the δ2D database yields the secondary structure propensity of the protein. **(A)** Helix propensity. **(B)** Extended beta sheets propensity. **(C)** Random coil propensity. **(D)** Polyproline II propensity. Residue numbers are those of pdb id:2FQL.

The analysis of the two assignments is nicely complemented by the use of a newly developed program, δ2d (Camilloni et al., 2012), which in addition to canonical elements of secondary structure yields also polyproline II propensities for each residue. The graphs of Figure 7 show that the divergence between the assignment of the present work and the previous one (BMRB accession code: 6356) is even more pronounced. The region where the divergence occurs maps onto a highly negatively charged region which contains a network of spatially nearby uncompensated negative charges, originating from the side chains of E89, E103, D101, and E112 (Figure 6C). According with the presence of a highly frustrated region, the extent by which this region is influenced by salt is clearly observable in the SSP plot. The corresponding residues in other tested frataxin orthologs are not equally charged, correlating with their higher stabilities and with the ability of Yfh1 to undergo cold denaturation. These data thus strongly suggest the presence of energetic frustration due to the high density of negatively charged residues as a major cause of Yfh1 instability.

## Discussion

The stability of globular proteins reflects the subtle balance of several different stabilizing and destabilizing contributions. Usually, we assume that proteins have evolved to optimize their stability with respect to the environmental constraints which they are active in, among which temperature. Proteins from mesophilic organisms, for instance, are usually stable around room temperature and over a relatively large range of temperatures (Razvi and Scholtz, 2006). There are however, examples of globular proteins which are just about stable also at room temperature. Yfh1 is not the only example of such behavior: while the fold of many globular proteins is unaffected by changes in experimental conditions within a large temperature range, other proteins, such as the well characterized U1A (Ternström et al., 1999) and azurin (Zong et al., 2007), have a broad transition energy barrier which leads to what we could call a “malleable” structure. This behavior was suggested to arise from the presence of strained energetics in the native state ensemble which leads to energetic frustration (Rimratchada et al., 2014). The systems that experience such frustration may provide unique information on both the early and late events of folding, as well as on the continuum of states in between. They are thus of particular interest from an experimental perspective.

For marginally stable proteins, a fascinating question is whether their behavior is evolutionary selected and beneficial for their functions or if other factors might intervene to stabilize the proteins so that they do not usually experience *in vivo* the conditions under which they are unstable. To understand the determinants of Yfh1 instability, we have analyzed in detail the behavior of the protein at room temperature. In previous studies we identified both the length of the C-terminus (Adinolfi et al., 2004) and salts (Sanfelice et al., 2014) as important factors that influence the stability of Yfh1 to the point that most salts increase considerably the unfolding point. Here, we have proven that Yfh1 exists in solution at room temperature as an equilibrium mixture of a folded and a partially unfolded species and that the equilibrium can be influenced by variations of the ionic strength. At low ionic strengths, the two species are present with an approximately 70-to-30 folded/unfolded ratio in favor of the folded conformer. The equilibrium is completely shifted toward the folded species when the ionic strength is increased. The intermediate has a spectrum broadly reminiscent of the spectra observed in the unfolded states at high and low temperatures (Adrover et al., 2010, 2012; Sanfelice et al., 2014). We believe that this is currently the only example that permits a comparison between NMR spectra of genuine unfolded or partially unfolded species at three different temperatures for a non-intrinsically unfolded protein.

Further, we mapped the effects that control the position of the folded vs. unfolded conformational equilibrium of low ionic strength Yfh1 to a specific region of the protein. Through the chemical shifts of the folding intermediate, we have experimentally identified the region of the protein where unfolding occurs (this study). The experimental evidence points toward a small cluster of negatively charged residues spatially close in the structure as an important source of energetic frustration. They comprise D101 and E103 in the β 1 strand and E112 in β 2 and form an “electrostatic hinge.” An important contribution of the β-sheet in destabilization is also fully consistent with previous indications that this region is the most affected by addition of mono- and di-valent cations with the consequence of stabilizing the protein up to rendering the stability curve of the protein much more shallow and to shifting cold denaturation to much lower temperatures (Sanfelice et al., 2014). This is explainable considering that proteins are polymers with many degrees of conformational freedom whose internal repulsive energetic interactions are typically screened to small distances. However, the residues of Yfh1 are so close in space that can only be efficiently screened by counterions. These residues are semi-conserved in other members of the frataxin family correlating well with the somewhat unusual instability of Yfh1 as compared to other orthologs (Sanfelice et al., 2014).

Our results have important implications. On a practical side, they stress the importance of salt to carry out *in vitro* functional studies aimed at characterizing the native function of proteins and specifically of Yfh1. More importantly, they indicate the importance of cation binding for the fold and function of Yfh1. In facts, the residues involved are among those identified to participate in iron binding and in recognition of one of the important natural partners of frataxin, the desulfurase Nfs1 (Adinolfi et al., 2009). We can also predict that the destabilizing interactions are where unfolding starts and that they are an important factor for observing cold denaturation of this protein. It will be interesting in the future to mutate some of these residues to release the effect and analyze how this may affect fold stability.

Finally, it is in order to draw a comparison between Yfh1 with another prototypical example of marginally stable proteins stabilized by salts: this is the IscU protein which, interestingly, shares with Yfh1 the rather acidic isoelectric point (around pH 4.5), the intrinsic marginal stability that leads to cold denaturation at detectable temperatures and the existence of an equilibrium between a folded and a partially unfolded species in the absence of stabilizing cations (Iannuzzi et al., 2014). This analogy suggests that these are members of a new family of proteins in which energetic frustration due to destabilizing electrostatic interactions plays an important role in folding. It will be thus interesting to identify the causes that determine the properties of IscU at the residue level.

### Conflict of interest statement

The authors declare that the research was conducted in the absence of any commercial or financial relationships that could be construed as a potential conflict of interest.
