# Supplementary material for: Trapping a salt-dependent unfolding intermediate of the marginally stable protein Yfh1
Source: Front Mol Biosci. 2014 Sep 30;1:13. doi: 10.3389/fmolb.2014.00013 (PMC4428383; doi:10.3389/fmolb.2014.00013)
Supplement: Supplementary file 1 [file Presentation1.PDF]

Supplementary Information

Trapping a salt-dependent unfolding intermediate of the marginally stable protein Yfh1

Vilanova et al.

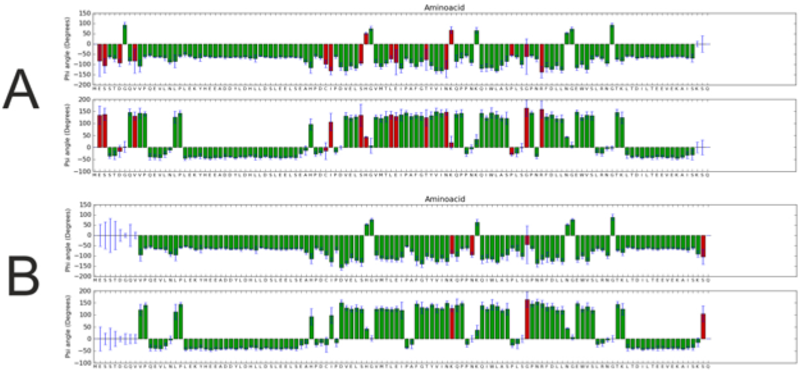

Figure S1. Talos+ analysis of chemical shifts. Chemical shifts from previous work A) (BMRB accession code: 6356) and the new one (BMRB accession code: 19991) were analysed using Talos+ Server (http://spin.niddk.nih.gov/bax/nmrserver/talos/). Green bars refer to residues that are in a favorable region for angles φ and ψ, red are not allowed regions and were the bars are missing, it refers to flexible residues.
